# Diagnostic accuracy of antibiograms in predicting the risk of antimicrobial resistance for individual patients

**DOI:** 10.1017/ash.2023.276

**Published:** 2023-09-29

**Authors:** Shinya Hasegawa, Jonas Church, Eli Perencevich, Michihiko Goto

## Abstract

**Background:** Many clinical guidelines recommend that clinicians should use antibiograms to decide on empiric antimicrobial therapy. However, antibiograms aggregate epidemiologic data without consideration for any other factors that may affect the risk of antimicrobial resistance (AMR), and little is known about an antibiogram’s reliability in predicting antimicrobial susceptibility. We assessed the diagnostic accuracy of antibiograms as a prediction tool for *E. coli* clinical isolates in predicting the risk of AMR for individual patients. **Methods:** We extracted microbiologic and patient-level data from the nationwide clinical data warehouse of the Veterans Health Administration (VHA). We assessed the diagnostic accuracy of the antibiogram for 3 commonly used antimicrobial classes for *E. coli*: ceftriaxone, fluoroquinolones, and trimethoprim-sulfamethoxazole. First, we retrospectively generated facility-level antibiograms for all VHA facilities from 2000 to 2019 using all clinical culture specimens positive for *E. coli*, according to the latest Clinical & Laboratory Standards Institute guideline. Second, we created a patient-level data set by including only patients who did not have a positive culture for *E. coli* in the preceding 12 months. Then we assessed the diagnostic accuracy of an antibiogram for *E. coli* to predict resistance for the isolates in the following calendar year, using logistic regression models with percentages in the antibiogram as dependent variables. We also set 5 stepwise thresholds at 80%, 85%, 90%, 95%, and 98%, and we calculated sensitivity, specificity, and accuracy for each antimicrobial. **Results:** Among 127 VHA hospitals, 1,484,038 isolates from 704,779 patients were available for analysis. The area under the ROC curve (AU-ROC) was 0.686 for ceftriaxone, 0.637 for fluoroquinolones, and 0.578 for trimethoprim-sulfamethoxazole, suggesting their relatively poor prediction performances (Fig. 1). The sensitivity and specificity of the antibiogram widely varied by antimicrobial groups and thresholds, with substantial trade-offs. Along with AU-ROC, these metrics suggest poor prediction performances when antibiograms are used as the sole prediction tool (Fig. 2). **Conclusions:** Antibiograms for *E. coli* have poor performances in predicting the risk of AMR for individual patients when they are used as a sole tool, and their contribution to the clinical decision making may be limited. Clinicians should also consider other clinical and epidemiologic data when interpreting antibiograms, and guideline statements that suggest antibiogram as a valuable tool for decision making in empiric therapy may need to be reconsidered. Further studies are needed to evaluate the contribution of antibiograms when combined with other patient-level factors.

**Figure:**
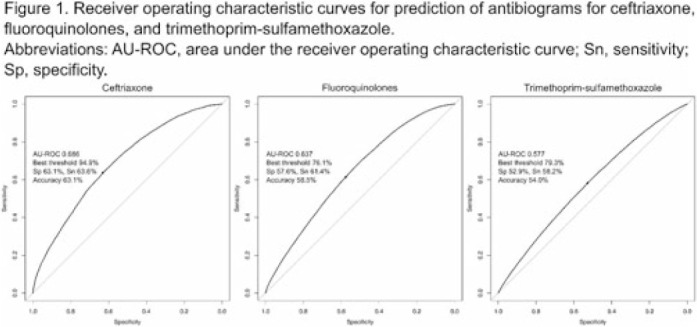

**Figure:**
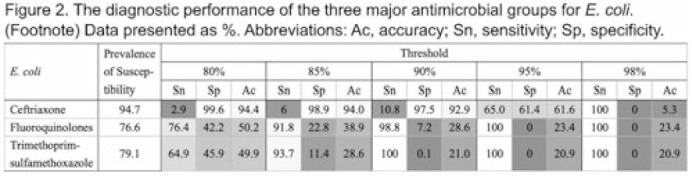

**Disclosures:** None

